# Safety and Health Measures for COVID-19 Transition Period in the Hotel Industry in Spain

**DOI:** 10.3390/ijerph18020718

**Published:** 2021-01-15

**Authors:** Rafael Robina-Ramírez, Jose-Amelio Medina-Merodio, Libertad Moreno-Luna, Héctor V. Jiménez-Naranjo, Marcelo Sánchez-Oro

**Affiliations:** 1 Department of Business and Sociology, Universidad de Extremadura, 10071 Cáceres, Spain; limorenol@alumnos.unex.es (L.M.-L.); msanoro@unex.es (M.S.-O.); 2 Department of Computering Science, Universidad de Alcalá, 28801 Alcalá de Henares, Spain; 3 Department of Finance and Accounting, Universidad de Extremadura, 10071 Cáceres, Spain; hectorjimenez@unex.es

**Keywords:** COVID-19, tourism industry, transition period, health authorities, pandemic, hotel managers

## Abstract

The health crisis caused by the outbreak of the COVID-19 disease has devastated the worldwide hospitality sector. The current situation has led many countries to implement drastic rules to stop the spread of the virus. According to the Spanish health authority decisions need to be made in the context of uncertainty and lack of knowledgeable experiences through a gradual and asymmetric de-escalation process planned in four phases. Although the vast majority of studies refer to economic risks and impacts on tourist flows and economic income, few of them explicitly investigates safety and health measures that hotel managers should implement to their customers. Over a population of 12,740 hotels, 823 Spanish hotel managers have been involved in a participatory study. With the aim of assessing the actions taken to stop the spread of the virus, empirical research was implemented. A model presented four variables and 13 indicators which have been previously tested among hotel managers in the tourism sector. Five conclusions are drawn from the hypotheses: (1) Mass testing surveillance in customers and employees should be quick, affordable, and homogeneous throughout the European Union. (2) Training measures need to be taken by both public authorities and the private sector to reach a knowledgeable crisis management team with high commitment to the customer’s health and safety. (3) Protocols established by public authorities should be observed and adjusted gradually not only in hotels but also in tourist arrivals. (4) Healthy measures need to be periodically updated. (5) Each hotel should set up a surveillance process to guarantee the safety to their customers.

## 1. Introduction

As Spain is the second most popular tourist destination in the world [1], the COVID-19 epidemic has had a devastating effect on the country with more than 27,709 deaths [2]. In this scenario, the tourism industry has been severely harmed as a result of the lockdown to contain the outbreak [3]. It is an unprecedented situation in which global tourism has moved from overtourism to nonexistent touristic activities [4].

In the last two decades several studies have addressed warnings about the role that pandemics play against the tourism sector [5]. To understand the relationship between tourism and pandemics, it is crucial to delve into the role that health security plays in global crisis, not focused on territorial impacts but on international ones [5]. Few studies have addressed this perspective analyzing the impact of air on propagating the coronaviruses [6] and the role that travel plays in relation to the epidemiology and disease surveillance [7,8].

Under the current damaging situation for the tourism sectors, scholars are currently proposing global solutions to flatten the curve by reducing the number of cases and its effect on the tourism industry [9,10,11,12,13,14]. Although the recovery process of the hospitality sector from the COVID-19 disease is currently under construction through theoretical proposals [15,16] empirical research is scarce [17] and some solutions have started to be proposed. Among these, the new outbreak of the COVID-19 virus has led many countries to implement drastic restrictive measures [18]. These proposals include closing public spaces, restaurants, and schools as well as restricting any economic activity that induces close physical contact between workers [19].

The European Commission through the document “COVID-19: EU guidance for the progressive resumption of tourism services and for health protocols in hospitality establishments” has established criteria and recommendations for the Member States on the conditions to lift the measures and restore free movement [20]. These criteria have a direct impact on tourism companies since the protection of the health of citizens remains the key priority, including tourism workers and tourists [20].

Since the epidemic peaked in Spain in the last week of April 2020, health authorities have established plans for a gradual transition towards the opening of bars, hotels, and restaurants [21]. However, the little preparedness in the tourism industry combined with doses of uncertainty and lack of knowledgeable experiences about how to face that negative impact has recently not been helpful to the Spanish tourism industry [22].

In the current situation of uncertainty, solutions have been implemented during previous pandemics in the tourism industry [23]. The Institute for Spanish Tourist Quality (ICTE) is developing a draft document with 21 measures to protect workers and tourists. These measures seek normalization to generate confidence in European tourism [24]. Those changes are developing to generate trust among customers in the area of health protection either for customers or employees, strategical technology plans, and post-lockdown strategies [25].

The vast majority of studies refer to economic risks and impacts on tourist flows and economic income [26,27]. Among them economic and financial activities have been classified in policies to reduce the COVID-19 damaging effect [28].

Recent studies have analyzed the social cost and benefits for destination communities in general [29] and destination residents during the COVID-19 crisis [30]. Nevertheless, few of them have explicitly investigated the measures that hotel managers should implement to offer safety and health to their clients [31]. As far as we know, there is no research focused on the tourism companies to stop the spread of the virus.

**Figure 1:**
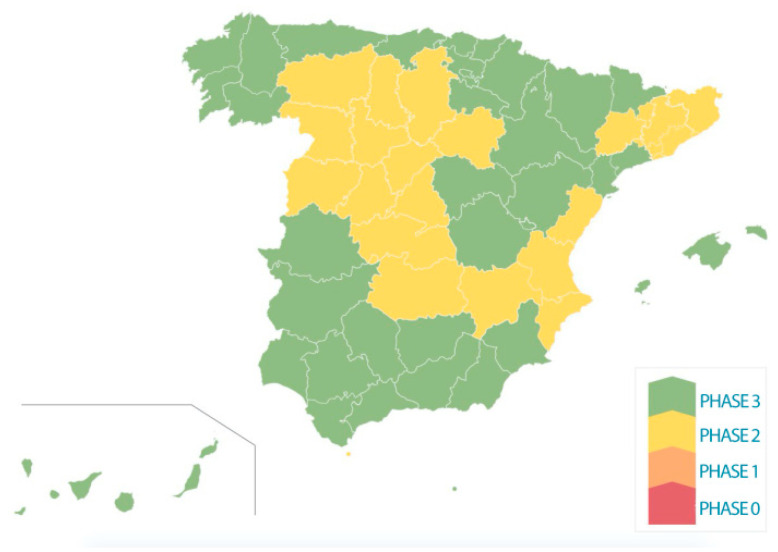
De-escalation process in provinces on 30 June 2020. Notes: The activities allowed in each phase are conveyed in the notes below.

Unlike other European countries, such as France and Italy, which have announced a schedule of restrictive measures to implement for the reopening of stores and businesses, Spain has used the term ‘phases’ to be applied in provinces with a low to high rate of infection (Royal Decree-Law 16/2020). In these locations, the de-escalation will be gradual and asymmetrically planned in phases in which movements between provinces or islands are not allowed. From phases 0 to 3, tourism will reach the normal situation that existed prior to the pandemic (Royal Decree 394/2020 of July). Figure 1 shows the de-escalation process during the data collection in June and July. The criteria the Spanish government have used to decide what territories can change from one phase to the next one are the capacity of the health system, the epidemiological situation in the area, the protection measures in public spaces, and the mobility and socioeconomic data of islands or provinces.

Phase 0. Opening of some business (only with previous appointment); restaurants with food delivery service or hairdressers, among others; common relief measures such as letting children go out to play for an hour, individual sports and family walks.

Phase 1. Mobility: possibility of moving within the same province; social gatherings of up to 10 people in private spaces are allowed, always respecting the physical distance; activities in stores will begin in the small shop “in conditions of strict security”; open-air markets, with distance conditions between the stalls; bars and restaurants: the opening of bar and restaurant terraces at 50% of their capacity will be allowed; hotels and tourist accommodation: opening of hotels and other tourist accommodation, excluding common areas and with a preferential schedule for people over 65 years of age; cultural shows of less than 30 people indoors (with a third capacity) and less than 200 people outdoors; visits to museums limited to one-third of the capacity; agri-food and fishing sector: the resumption of agri-food activity that had been stopped in the decree of the state of alarm will begin; places of worship: they can open with their capacity limited to one third.

Phase 2. Opening of bars and restaurants for table service, with limited capacity; trips to second residences, only if they are in the same province; reopening of shopping malls, prohibiting the stay in common areas or recreational areas; cinemas and theatres with a third of the capacity; cultural activities with less than 50 people seated indoors; educational centers: they may open for reinforcement activities, to ensure that children under the age of six can go to the center if both parents have to work and to ensure the “Selectividad” exams take place, as that is a compulsory exam to access university; weddings for a limited number of attendees; places of worship: the capacity is limited to 50%; hunting and fishing.

Phase 3. General mobility will be made more flexible; the use of masks in public transport will continue to be recommended, as in all previous phases; stores: capacity will be limited to 50%, with the requirement that there be a minimum distance of two meters between people; bars and restaurants: capacity restrictions will decrease but always maintaining the separation conditions between clients; discos and night bars with a maximum capacity of one-third of the usual; opening of beaches in safety and distance conditions. Bullrings: with a capacity limitation that guarantees one person for every nine square meters.

Following a participative methodology, this paper focuses on analyzing what safety and health actions should be taken to stop the spread of the virus in the tourism industry. Due to the ongoing COVID-19 pandemic in Italy in February 2020 and the lockdown in Spain from March to May, a group of Spanish hotel managers decided to collaborate on the study during June and July to delve into its potential consequences in the Spanish tourism industry.

Out of a population of 12,400 hotels in Spain [2], 823 hotel managers were involved in the study. Following a participative methodology, two focus groups were organized to study the hotel managers’ perceptions about the effect of the pandemic in the tourism industry. Twenty-three hotel managers were involved in the meetings in the first half of June 2020. Safety and health drivers for the COVID-19 transition period were drawn from the literature review, as well as the items that define those drivers, given the previous experience of tourism managers in crisis management in global tourism [32].

The contribution of this research is twofold. First, following the Structural Equation Model (SEM-PLS) a model of actions to preserve companies from the spread of the virus is presented. Second, hypotheses are proposed from the tourist providers’ perspectives to analyze its correlation and significance as insightful paths to be observed by the tourism industry. The document begins with a brief review of stages of a crisis in the context of the restrictive measures announced by the Spanish Minister of Health. Next, the restrictive measures are studied under the umbrella of the tourism industry. The methodology used in data collection, analysis of results, discussion, and conclusions complete the sections of the work.

## 2. Materials and Methods

Since the outbreak of the COVID-19 pandemic, hotels, travel agencies, and the media and professionals of the touristic sector in Spain have been warning about the contagious risks of the virus in an interconnected world. However, these warnings were not appropriately followed by the Spanish Government. Delays in taking correct measures have ended up in health and safety crisis management to stop the spread of the virus. As a result, Spain became the second country to have the most cases at one point. Nevertheless, after closing its borders and making repeated announcements to “stay at home”, the disease appears to have been apparently reduced.

In this new scenario, close collaboration between the public and private sectors has become crucial, as a combined strategy, health authorities and the tourism sector, to appropriately face the new pandemic.

Several safety and health drivers for crisis planning and management by public–private sector organizations are described in the next sections. Among them, the role of social distancing in the tourism industry, the attention to sanitation measures, and mass testing and technology are playing a pivotal role either for the public or private sector in the tourism industry.

### 2.1. Stages of the Epidemic Crisis. Drivers for COVID-19 Transition Period (C19TP)

As Ritchie [33] explained, the range of crises in organizations can scale from minor problems, such as staff illnesses, to massive business disruptions due to pandemics and natural disasters. This range can be organized in phases depending on the damage they cause to the organizations [32]. Commonly, cases involving pandemics and disasters are defined in four stages: pre-crisis, crisis, post-crisis, and long-term recovery [34]. From another perspective, Faulkner [35] analyzed crises starting with an emergency phase, with no return point; an intermediate phase, in which recovery will possibly take several years; a long-term phase in which the economy and the market will be restored in a short period of time. The time scale involved in each stage may require different responses and strategies to plan and manage the results [36]. The transition period between each stage can lead to a worsening or a recovery stage [37].

Governments are preparing contingency plans with proactive and strategic approaches based on crises prevention to revive the tourism industry [38]. Cutter [39] proposed three different scenarios: (a) maintenance of essential services to reduce the effect on the economy and social life of the country; (b) extend antiviral treatment to reduce morbidity and mortality; (c) increase the health services to slow down and limit the spread of the virus.

To reactivate the tourism industry, international organizations are developing new studies to analyze the role that the private sector plays in coping with pandemic COVID-19 [40]. As a result, managers have the responsibility to develop tools that they can use to prevent or plan for these crises and drivers to guide them during the transition period. Coordination between health authorities and companies is crucial to go further and take the right decisions to stop the spread of the virus.

In the United Kingdom, business organizations helped the Department of Health (2005) and the Government of Scotland has developed its plan and reaffirmed its leading role in this area [41]. This combined strategy-health authorities and the tourism sector- was used to define drivers stop the SARS pandemic and its influence on the tourism sector in the short- and medium-term [42].

Ritchie [16,17,18,19,20,21,22,23,24,25,26,27,28,29,30,31,32,33] described several drivers for crisis planning and management by public–private sector organizations that have three central components: prevention–planning, implementation, and evaluation–feedback. Likewise, other measures, such as border control, surveillance, information, education, communication (IEC), antiviral strategy, and vaccination strategy, should be implemented by the governments of the countries [43]. According to Bell [44], the effectiveness of these drivers during a pandemic has not been adequately evaluated during previous pandemics and the scientific evidence is limited.

### 2.2. The Role of Social Distancing in the Tourism Industry (SD)

The absence of available drugs and the discovery of a vaccine from 12 to 18 months in the future require companies to take social distancing measures [38]. According to the WHO [45], social distancing is one of the main key government measures to manage the COVID-19 crisis. This involves limiting contact between people to reduce viral transmission by maintaining at least 1 m (3 feet) of distance between two people as well as avoiding physical contact and ‘nonessential’ meetings of more than 500 people abroad and 100 people at home.

Social distancing limits the number of people who infect others through contact, even before they realize they have COVID-19 [46], to help to alleviate pressure on already overburdened healthcare and public health systems [47].

Comparing to other sectors, the tourism industry is more vulnerable to crises or disasters than other industries due to its high risk of infection between workers and tourists, along with repeated face-to-face meetings [48]. It affects the nature of policies and the evaluation process in order to adapt them to the severity of the pandemic [49].

In this scenario, private organizations should reduce physical interactions and take precautionary measures against contagion [50]. These measures include cancelling group meetings; conducting meetings virtually; keeping children away from group settings; promoting online connections between people or social networks to reduce the spread of COVID-19 [51].

In case of contagion, isolation or quarantine becomes another essential factor to be considered by companies [52]. To overcome those risky results, it is the responsibility of tourism companies to guarantee distance during the transition period, with clients being socially connected, whenever the client requests it [53]. In this context, organizations should analyze the services or products consumed by infected people to avoid the spread of the virus [54].

### 2.3. The Role of Attention to Sanitation Measures: Personal Hygiene and Hospital Infection Control (SHM)

Guidance on the provision of safe water, sanitation, and hygienic conditions has been offered by the WHO since the origin of the spread of the COVID-19 virus [38,39,40,41,42,43,44,45,46,47,48,49,50,51,52,53,54,55]. According to Luby et al. [56], handwashing reduced the incidence of respiratory infections in children and young adults in previous pandemics. In addition, many people wore masks, which helped prevent virus infection [57]. The use of protective measures plays an important role in offering tranquility to their workers [58].

Since Spain has moved into phases 1 and 2 [59], ICTE and main tourism stakeholders were responsible for developing guidelines to guarantee tourists sanitary and hygienic safety in hotels. These plans detail a whole series of measures to avoid infections, along with action protocols in the case infected people are detected in hotels.

### 2.4. Mass Testing and Technology (MT) in Hotels

The health authority in Spain has promoted the distribution of COVID-19 tests throughout the country to embark on mass testing to stop the contagious disease. The Spanish authority has also recommended hotels to use rooms, as quarantine centers, to house infected people with few or no symptoms.

As positive cases have been detected in the staff in Spanish hotels, COVID-19 tests are becoming mandatory in the hotels and recommended for tourists. Some hotels have imposed the obligation for guests to quarantine for 14 days after arrival or until they receive a negative COVID-19 test result. Data generated from tests provided by health authorities and business organizations [60] need to be communicated immediately to those responsible for the treatment and monitoring of the spread [61].

Initiatives to detect infections in companies are currently under development. The Johns Hopkins University Science and Systems Engineering Center has created a real-time monitoring map for following the cases of COVID-19 worldwide [62]. The installation of technological tools can also help in the early detection of outbreaks, allowing public safety to be maximized [63]. The tools include thermal cameras or Internet of Things (IoT) sensors. These sensors would allow tourists to take temperatures in addition to perceiving those who have developed antibodies against the new coronavirus COVID-19 [64]. The IoT devices must support open protocols and, at the same time, the device provider must ensure that data integrity and security are respected during communication and transmission [65].

Actions like this would slow down propagation of the virus and reduce the risk of contagion among tourists from different parts of the world in which it is still active [66,67]. However, accessing data for many companies is a challenge because the information is often considered sensitive for national security reasons, acknowledging that a virus outbreak is an equal threat to national security and the economy.

## 3. Methodology

### 3.1. Methodological Framework

Since the first week of June, the lockdown measures in Spain keep on easing thanks to positive results of the past weeks in terms of contagion and disease. This means that more social and economic activities were operational again, although at low levels. The hospitality industry was able to open their touristic activities but with limitations for common areas.

To measure the perception of hotel managers about the current security and health system, the research team contacted with the 19 tourism boards spread among the autonomous regions in Spain in the first week of June by email and phone calls.

The first aim was to get the updated contacts of Spanish hotels. Since the first calls the research team had little responses from the boards, they kept trying until reaching the response from 17 of them a week afterwards. There were no responses from Ceuta and Melilla on the northern shores of Morocco’s Mediterranean coast. The list of hotels and contact details reached 12,740 hotels in total. The data was contrasted with statistics provided by the Spanish National Statistics Institute [2]. Table 1 shows the population and the sample of hotels in the study in each region.

According to the Spanish National Statistics Institute [2] there are two categories in Spanish hotels: “Gold” for hotels, hotel-apartments, hotel-residences, “Paradores Nacionales” and residence-apartments. The second category is called “Silver”. It is for hostels, guest houses, and others. Table 2 shows the hotel distributions according to the two touristic categories in Spain.

The epidemic prevention by the government has been based on hygienic-sanitary recommendations for medicalized hotels and hotels open for essential services released by the Spanish society of public health (SESA) and health administration (SEMPSPH). In view of the emergency produced by the COVID-19 pandemic and the declaration of a state of alarm by the Government of Spain, although tourist accommodation establishments were closed to the public, it was also decided that some of them were considered essential services to provide accommodation coverage for “professional activities of an essential nature”. In addition, the Health Authority has also considered the convenience of converting some hotel establishments into medicalized hotels to accommodate patients affected by COVID-19. For these two types of hotels, the Spanish Society of Environmental Health in collaboration with the Spanish Society of Preventive Medicine, Public Health and Hygiene have prepared a Guide with the set of hygienic-sanitary recommendations that should be considered in the two types of hotels to guarantee that clients and patients are not affected by risks of environmental origin. Thus, the emphasis has been placed on the quality of the water and the prevention of any viral disease; air quality, food safety, and health. With the SESA initiative public health has contributed to the resolution of this pandemic and not only the assistance devices or epidemiological surveillance. In the case of developing contingency plans for new epidemics, professionals of either Environmental Health or Food Safety, will apply their knowledge and experience to successfully address complexity, uncertainty, and ambiguity of public health risks. Table 3 shows the medicalized hotels and hotels open for essential services distributed according to the hotel touristic categories.

Even though there has been no difference between hotels’ touristic categories, the distributions of these hotels amongst the Spanish autonomous regions has been released. Table 4 shows that distribution.

### 3.2. Variables and Questionnaire

From the list of Spanish hotels, we randomly selected and contacted three hotels from every Spanish tourism board at the end of the first week of June to participate in two zoom meetings. A letter was sent to all of them to explain the aim of the research as well as the methodology. A total of 23 out of 51 were willing to be involved in the study. In order to debate with these hotel managers about the drivers for the COVID-19 transition period, a list of items drawn from the literature was proposed.

According to these items, in the first meeting, an interactive debate between hotel managers involved in the research provided detailed information in relation to the proposals offered by experts in the area of health. Four topics were set on the table to be discussed and compared with the original items.

- To involve hotel managers in building protocols by designing mechanisms for gathering participative information to make the best decisions, especially by creating protocols to identify possible symptoms caused by the virus such as implement a temperature control system, ventilation systems, and other hygiene and safety measures.
- To develop and implement a contingency plan by offering an agreement between hotels and hospitals to offer tourist 24/7 medical care and insurance services as way of building confidence and trust. It includes flexible upgrades to offer bigger rooms in case of unexpected quarantines.
- To introduce flexibility measures in cancellation policies and develop a plan for customers to avoid public transportation by ensuring transportation from the airport to the hotel.
- Keep the team updated about the evolution of the virus in the tourist destination involving staff members in the actions taken to prevent the virus.

The debate was especially around how to identify the existing risks in the provision of certain tourist services, what mechanisms exist to gather information that allows them to make the best decisions to adopt in the future transition period, what contingency plan should be designed to consider various phases of evolution in relation to its stakeholders (service providers, authorities, other guides, etc.), and how to assess that contingency plan and its effectiveness.

According to the results of the debate, four latent variables were proposed: C19TP: drivers for COVID-19 transition period; SD: social distancing; SHM: sanitation and healthcare measures; MT: mass testing.

In the second meeting, during the second week of June, the changes in the original items were introduced. After an intense debate, all the items were modified and expanded by the hotel managers (see Table 5).

Each item was then formulated into question mode. The questionnaire had been previously validated through 10 qualitative interviews conducted with hotel managers who were different from the participants in the two focus groups.

In the second half of June 2020, mass mailings were sent to hotels with a link to a Google doc to which the questionnaire was attached. At that time, national authorities started announcing the end of the state of alarm in Spain, which came to an end on 21 June.

The questionnaires were distributed using Free Online Surveys. To avoid missing data the questionnaire was divided in five sections. To move from one section to the next one hotel manager had to match all the responses. Some of them were fulfilled by phone. Therefore, the research team was especially focused on gathering all the responses from the managers.

The research objective of the academic group was to obtain the maximum number of responses from hotel managers. Initially, the research team decided to carry out a stratified sampling based on the hotel’s tourist categories. From there, the same number of questionnaires was selected, 100 for each touristic category were sent by emails to hotel managers from five-star hotels to one-star hotels with gold or silver categories. At the end of the sampling period, three conclusions were drawn: (1) Almost 15% of the emails had wrong address. A total of 90% of those wrong addresses were updated. (2) Differences were found between five-star and four-star hotels’ responses compared to three-star, two-star, and one-star hotels. (3) The number of total responses was very low to draw significant conclusions.

After these results, the research team decided to extend the sampling to the entire population by sending emails in groups of 50 hotels. To introduce the study to them messages were organized according to the hotel’s tourist category.

Of the emails extended to the whole population, 20% were returned due to the wrong addresses and 35% did not respond. Of the other 45%, only 4% approximately responded favorably, 560 questionnaires through Google Forms were collected. In the next week 300 phone calls inviting to hotel managers to complete the questionnaire were undertaken in the last week of June. However, 17 were matched wrongly. The final sample of participating hotels was 843.

### 3.3. Hypotheses and Model

In accordance with the four constructs designed by the hotel managers, the hypotheses ascertained the relation of the main drivers of hotel managers and the exogenous variables (SHM; MT; SD). The drivers are focused on three items:

1. The effect of reopening borders to the tourism sector by providing safe and healthy solutions to tourists.
2. The development of protocols for the gradual transition period according to the health authority.
3. The installation of affordable solutions for the tourism sector to open the hotels in safe conditions.

**Hypothesis 1 (H1):**  *Sanitation and healthcare measures (SHM) positively influence the drivers for the COVID-19 transition period (C19TP).*

**Hypothesis 2 (H2):**  *Sanitation and healthcare measures (SHM) positively influence social distancing (SD).*

**Hypothesis 3 (H3):**  *Social distancing (SD) positively influences the drivers for the COVID-19 transition period (C19TP).*

**Hypothesis 4 (H4):**  *Sanitation and healthcare measures (SHM) positively influence mass testing (MT).*

**Hypothesis 5 (H5):**  *Mass testing (MT) positively influences the drivers for the COVID-19 transition period (C19TP).*

**Figure 2:**
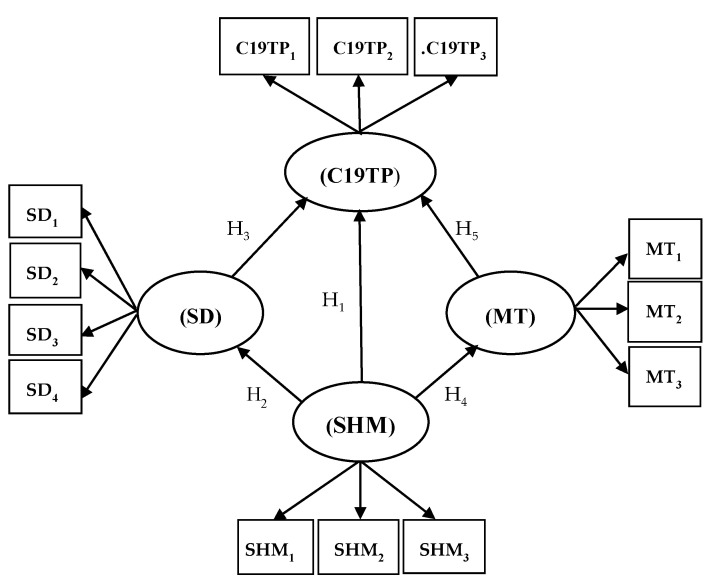
Research model designed. C19TP: drivers for COVID-19 transition period; SD: social distancing; SHM: sanitation and healthcare measures; MT: mass testing.

The proposed theoretical model is shown in Figure 2.

The information obtained was processed following the parameters of the structural equations (SEM). This statistical technique is observed when dependency relationships are established between latent variables and indicators [73].

For the generation of the statistical model, the PLS (partial least squares) technique applied was SmartPLS 3 Version 26 (SmartPLS GmbH, Boenningstedt, Germany). This version is especially recommended for composite models [74].

## 4. Results

### 4.1. Results of the Measurement Model

SEM-PLS modeling was defined based on two approaches: the measurement model and the structural model. To proceed to the analysis of the structural model, it was necessary to analyze the reliability that exists between the indicators and the constructs as well as the validity of the measurement model [75]. In this case, we used reflective elements because they are interchangeable [76]. Reliability was studied in Table 6 by analyzing individual loads or simple correlations of the measures with their respective latent variables, ≥0.7 was accepted [77].

Cronbach’s alpha coefficient and composite reliability were also used as the reliability index of the latent variables. The convergent validity of the latent variables was analyzed through the extracted average variance (AVE) (accepted >0.5). To study the discriminant validity of the latent variables, the Fornell–Larcker criterion was used [78]. This criterion examines whether the square root of the average value extracted (AVE) of each item is greater than the correlations with the other latent variables, as shown in Table 7.

According to [79], it is necessary to implement techniques that better detect the absence of discriminant validity. In this case, the test that was applied is called the heterotrait–monotrait ratio (HTMT). If the relationship for each pair of factors is <0.90, the condition is accepted [80]. Table 8 shows the valid values for the HTMT test.

### 4.2. Results of the Structural Model

After examining the measurement model, we moved on to the structural model. For this, the path coefficients of each of the hypotheses were studied. To obtain these values, a starting programme of 5000 subsamples was applied to verify the statistical significance of each route.

The overall fit of the model was evaluated using the standardized mean squared residual standard indicator (SRMR) [81]. SRMR is the average difference between the predicted variances and covariance and those observed in the model. Therefore, a small value reflects a good fit. A cut-off value of 0.08 is considered the most appropriate [82]. In the study, the SRMR was 0.076, which means that the model fits the empirical data [81].

The explained variance (R^2^) of the endogenous latent variables and the *p* value of the regression coefficients (*t*-test) were used as indicators of the explanatory power of the model [83]. The R^2^ values maximize the amount of explained variance obtained for the investigation and led to the following conclusions: 0.67 ‘Substantial’, 0.33 ‘Moderate’, and 0.19 ‘Weak’ [84]. The result obtained explains the positive relationship between the measures to be taken by hotel managers to protect the health and safety of their clients. The amount of variance explained by the drivers for the COVID-19 transition period (C19TP) is R^2^ = 52.6%. The evidence, therefore, shows that the presented model has a moderate predictive capacity. This explains the reason the variables of social distancing (SD), sanitation and healthcare measures (SHM), and mass testing (MT) become key factors in introducing the implementation of security and hygiene measures in hotels during the transition period (C19TP).

Table 9 shows the results obtained that allowed us to accept all the hypotheses since there were no statistically significant differences in the relationships between the variables in our model (*p* value > 0.05).

Geisser [68,69,70,71,72,73,74,75,76,77,78,79,80,81,82,83,84,85] and Stone [86] recommended evaluating the Stone–Geisser test as a criterion for evaluating the predictive capacity of the model (Q^2^). To determine this in SmartPLS, it is necessary to generate the blindfolding procedure. After the Stone–Geisser (Q^2^) test [85,86], the values were as follows: 0.02, 0.15, and 0.35, which indicates small, medium, and high predictive relevance, respectively. As a result, Table 10 shows that endogenous constructions meet (Q^2^) >0.

## 5. Discussion

The discussion section is organized in three parts. First, according to the model presented a comparative result is highlighted. Second, critiques to the pandemic crisis carried out by the Spanish government. Third, hotel managers’ health and safety perceptions depending on the type of hotel.

### 5.1. Comparative Results Drawn from the Model

The world is currently in the midst of a state of pandemic crisis with devastating consequences for tourism [34]. In the case of Spain, the autonomous communities are going through different phases according to the epidemiological situation each territory is going through. Considering that each phase requires different strategies [36], collaboration between the public and private sectors is extremely necessary to revive the tourism sector [28].

The collaborative model presented in this work is based on a participatory study between hotel managers to implement public sector protocols in order to identify possible symptoms caused by the virus. As a result, “moderate-high” explanatory model with predictive capacity R^2^ = 52.6% is presented. The extended study among hotel managers allows us to address two of the three scenarios proposed by Cutter [39]. The collaboration of the private sector is channeled through the contribution to implement safe actions to open the hotels (C19TP3). According to Ritchie [33] those solutions provided by hotel managers to curb the spread of the virus would be prevention–planning (MT2), implementation–surveillance (MT3), and evaluation–feedback (MT1). Among those solutions, the possibility of expanding the capacity to test clients is especially relevant (SD3). Some hotels have signed an agreement with clinics to test clients for antigens, although this measure is still underdeveloped in most of the Spanish territories.

Hotel managers request the public sector to develop efficient actions to face the virus for the tourist arrivals at the hotels. Among them, guaranteeing the capacity and security of hospitals (SD1), controlling border health and safety (C19TP1), implementing new protection measures against new infections (SD2) such as surveilling the WHO policies; request the evaluation of safe water, sanitation, and hygienic conditions in hotels (SHM1) [56].

The predictive relevance (Q^2^) (C19TP = 0.381), (MT = 0.205), (SD = 0.204) allows to replicate the research model in other sectors [48], with high degree of contagion between workers and clients, such as the health or agri-food sector where it is necessary to exercise extreme caution to stop the spread of the virus [50].

### 5.2. Critiques to the Pandemic Crisis Carried Out by the Spanish Government

From hotel managers’ surveys, open questions were collected through Google Forms and through phone calls. Common disagreement with the health authority was expressed. The criticisms were focused on four areas: (1) Mobility restriction and economic consequences. (2) Security protocols. (3) Digitization and new technologies. (4) Testing measures.

Statements such as “opening a hotel with severe restrictions on both mobility and capacity would be unfeasible because it could lead to bankruptcy” were criticized in the interviews (3, 7, 23, 32). Some hotel managers asked about “who is going to stay in our establishments? Our neighbors in the area? It is not even allowed to travel even between different provinces” (interviews 18, 27, 33). Others identified the “restrictions with the necessary generation of profit; they said that anyone who has an idea of hotel equipment knows that opening a hotel at 30% means losing money. In order to cover costs, it would have to be at 50% and from then on it would see the benefits and capacity limitations” (interviews 5, 14, 15, 34). Criticism went also around the “lack of understanding about why their hotel establishments are limited in capacity when other sectors, such as, for example, supermarkets or pharmacies, are not” (interviews 16, 25, 31).

In relation to security protocols, not only did they say that it is ‘necessary to work on security protocols to guarantee health for our customers’ (interviews 1, 6, 27), but also these ‘protocols have not yet been communicated to them by the health authorities and they have not yet received health guidelines to follow’ (interviews 12, 19, 21, 24). Then, they ‘demand more information to know how to comply with the rules of distancing and health depending on the type of premises’ (interviews 13, 17, 29, 30). Given this lack of information, hoteliers’ associations are proposing concrete measures; they believe that it is the private sector that has to propose the measures to policy makers. Furthermore, ‘they advocate a single health protocol for all of Spain and not that each region has its own’ (interviews 7, 26, 28, 32).

Health protection also involves the implementation of digital technologies as established by the European Commission [25]. It does not only mean the access by tourists to information on borders and travel, health, and safety conditions in establishments [63]. It is also the use of mobile applications, artificial intelligence (AI), and robotics to monitor the planning of physical distancing in accordance with the data protection law [66] and management of the flow of tourists as well as disinfection and hygiene through the use of robots [63,64]. As a result, ‘the new protocols to avoid contagion will impose digitization, specifically nontactile activation methods’ (interviews 17, 23, 25, 27). ‘Without a doubt, the new technologies are helping them to implement many necessary protocols due to the expansion of the COVID-19′ (interviews 2, 4, 11, 12).

In relation to testing measures: ‘we will carry out quick tests on our clients through noninvasive temperature controls so that we can guarantee no infections in the hotel’ (interviews 8, 13, 14, 33).

### 5.3. Health and Safety Perception Depending on the Type of Hotels

According to a survey carried out by the DNA (Tourism and Leisure consultancy) during April and May, more than 80% of Spaniards considered the option of traveling despite the shutdown. Over 70% wanted to travel accompanied by their partner or direct family, 30% with friends. The vast majority rejected crowded destinations, preferring the isolated destinations [70]. Therefore, what they value most was safety and health, even more than the price. That perception varied from ‘beach hotels’, ‘rural hotels’ and ‘city hotels’.

Currently, ‘tourists are fleeing from hotels in crowded cities and seek isolation. They prefer being in contact with nature than visiting beaches and big cities’ (interviews 12, 19, 21). However, some hotel managers have stated that ‘it will be impossible to abandon mass tourism at once, that’s why you have to find successful solutions to overcome the risk of contagion’ (interviews 4, 10, 15). As a result, hotel managers in coastal places ‘should be the first to react to overcrowded places by quickly publishing the protocols of the services, testing tourists and having a health passport’ (interviews 11, 35, 36).

On the contrary, rural accommodations have focused on small groups of visitors in isolated places. It should be the main reason to have increased the reservations during the pandemic crisis. ‘Since the government said on 28 April that rural establishments could open from 11 May, we began to notice an increase in reserves in the de-escalation period. They have risen significantly: 278% more than in the previous three weeks. Compared to last year we are returning to normal’ (interview 23). Moreover, rural accommodations, as small business, are closer to the clients and decisions are faster to implement than big hotels. ‘In our rural hotels, we evaluate daily the safety and hygiene measures in the rooms; in the breakfasts, wide distance and change of the buffet system; the partitions in the receptions; gloves, masks and hydroalcoholic gels scattered where necessary’ (interviews 11, 14, 15, 22). ‘In addition to the measures imposed by the health authority, we are going to do in our rural hotel everything we can think of, apart from thorough cleaning, we are ready to overcome every unplanned result to provide safety and health to our clients’ (interviews 9, 13, 16, 27).

Table 11 shows what relevance hotel managers give to the safety and health measures in relation to the three types of hotels. Rural hotels have conveyed slightly higher interest than beach and city hotels in providing these measures almost in every indicator. It might be explained due to the closer relationship with clients due to their small business in order to avoid the spread of the virus and their interest to protect their own small business. Most of them are directly managed by the owners.

## 6. Conclusions

Within the de-escalation phases proposed by the health authorities in Spain, it is necessary to guarantee health security in the tourist services that are provided to avoid the spread of the virus with new infections. Through a participatory methodology, hotel managers proposed a series of measures to be considered by these authorities. Three theoretical and four empirical conclusions are drawn from the results.

According to the results obtained from the path coefficients, all the hypotheses were accomplished, with high levels of significance. They were measured through Student’s *t*-test and *p*-values. Therefore, the relationships between constructs in the model presented are strong. This close relationship between the latent variables allows us to draw five conclusions ordered according to the size of the Student’s *t*-values.

The strongest relationship between variables corresponds to Hypothesis 4. It measures the incidence between sanitation and healthcare measures (SHM) and mass testing (MT) (SHM → MT; β = 0.534; T = 17.211). This relationship explains how health protocols proposed by the WHO to hotels affect the private sector’s responsible implementation. The enhancement of sanitary measures in hotels does not only refer to having a sanitary protocol of action approved by the private sector to prevent infections and relocate those infected, hotels must also develop their surveillance standards to avoid contagion, amongst them mass testing is the most valued. Hotel managers valued the ability to detect the employees and customers’ health status to avoid contagion. Aligned with these proposals, several interviews to manager directors show that tests should be quick, affordable, and homogeneous throughout the European Union (interviews 14, 16, 27). If those measures are effectively implemented in the hospitality sector, it would help to plan the tourist season for the coming months (interviews 4, 18, 25, 39).

The hotels’ second priority is conveyed in the Hypothesis 2, which shows the relationship between sanitation and healthcare measures (SHM) and social distancing (SD) (SHM → SD; β = 0.544; T = 16.815). The results covey that social distance and healthy measures in hotels have been shown as the most effective in preventing infections [29] which efficiently helps to reduce the number of infected patients in hospitals [30]. According to the hotel managers those measures have to be appropriately trained to reach a knowledgeable crisis management team to demonstrate high commitment to the customer’s health and safety (interviews, 1–6, 9–16, 18, 22, 28, 31).

The third conclusion is related to the influence of sanitation and healthcare measures (SHM) in the definition of the drivers for the COVID-19 transition period (C19TP) (SHM → C19TP; β = 0.353; T = 11.291) reported in Hypothesis 1. The current health and sanitary protocols established by public authorities should be observed and adjusted gradually without delay to the seriousness of the pandemic (interviews 4, 7, 10–18, 22–30). In order to curb a surge in coronavirus infections those measures should be applied not only in hotels but also in tourist arrivals in Spain. The control should be delivered by testing passengers from other countries, mainly from the risk ones (interviews 2, 5, 11, 16, 23, 29, 30)

The fourth conclusion refers to the relationship established in Hypothesis 3: social distancing (SD) influences the drivers for the COVID-19 transition period (C19TP) (SD → C19TP; β = 0.344; T = 8.157. Social distance and healthy measures need to be periodically updated, mainly for common areas as has been expressed by more than 90% of the hotels interviewed.

The fifth conclusion shows the influence of mass testing (MT) in drivers for the COVID-19 transition period (C19TP) (MT → C19TP; β = 0.177; T = 4.527). Every hotel has to deliver its own strategies to guarantee security and regain the trust of customers. These strategies include not only the availability of mass testing to customer but also setting up a surveillance process for staff shifts, daily individual protection equipment, disinfection of common areas and rooms, washing frequencies, distancing between reception and collection areas, food handling protocols, determining capacity in common areas, etc. (interviews 4–9, 12, 14, 15, 22, 28–32). Moreover, every hotel should collaborate immediately with the health and sanitation authority. A protocol to deal with this situation should be provided to customers. Some Spanish hotels are already offering this possibility through the checking-in, and they are also providing secluded rooms where private doctors perform viral tests for COVID-19 (interviews 3–11, 18–24, 32–34).

Two future research lines will be addressed. First, it is also relevant to consider the tourist authorities’ interests to introduce participatory methods to face the economic crisis with the tourism industry. Second, hotel managers’ behaviors will be studied to ascertain their level of agreement to develop the touristic decisions drawn from the potential participative programme. According to Schwartz [87], hotel managers’ attitudes, norms, responsibilities, and behavior will be analyzed. From Schwartz’s theory, personal beliefs, moral obligation, and behavior could be linked to the measures Spain needs to overcome the pandemic crisis [88].

## Figures and Tables

**Table 1:** Population and sample.

Spanish Regions	Population	Sample
Andalucía	2137	128
Cataluña	1889	119
Galicia	1150	51
Castilla y León	1143	80
Madrid, Comunidad de	1118	112
Comunidad Valenciana	884	63
Castilla-La Mancha	725	64
Aragón	703	39
Canarias	543	33
País Vasco	541	28
Asturias, Principado de	429	23
Extremadura	356	15
Cantabria	222	17
Navarra, Comunidad Foral de	219	8
Balears, Islas	141	19
Murcia, Region de	140	15
Rioja, La	130	9
Total	12,470	823

**Table 2:** Hotel distributions in Spain.

Categories	Hotels
Gold	
Five-stars	273
Four-stars	1859
Three-stars	1928
Two-stars	1559
One-star	961
Silver	
Three-stars	1581
Two-stars	1054
One-star	3255
Total	12,470

**Table 3:** Medicalized hotels and hotels open for essential services distributed according to the hotel touristic categories.

Medicalized Hotels	Hotels
Five-stars	39
Four-stars	127
Three-stars	121
Two-stars	71
One-star	2
Hotels open for essential services	
Three-stars	6
Two-stars	2
One-star	0
Total	368

**Table 4:** Medicalized hotels and hotels open for essential services distributed amongst autonomous regions.

Autonomous Regions	Medicalized Hotels and Hotels Open for Essential Services
Andalucía	51
Galicia	49
Castilla-León	36
País Vasco	35
Asturias, principado de	26
Aragón	25
Castilla-La Mancha	23
Comunidad de Madrid	19
Cataluña	19
Extremadura	17
Cantabria	16
Canarias	16
Baleares	14
Navarra	12
La Rioja	6
Ceuta	4
Total	368

**Table 5:** Preliminary study and list of items corrected by the managers.

Indicators	Original Items	Items Eventually Approved
(C19TP1) [68]	Tourists’ safety and security to slow down severe damage produced by the virus outbreak	Reopen borders to the tourism sector by providing safe and healthy solutions to tourists
(C19TP2) [21]	Gradual opening of bars, hotels, and restaurants	Developing protocols for the gradual transition period according to the health authority
(C19TP3) [69,70]	Measuring the impact of the health-related pandemic crises on tourism	The health authority should provide affordable solutions for the tourism sector to open the hotels in safe conditions
(SD1) [38]	Security and sanitation measures should be active until a vaccine is developed	Government should guarantee hospital capacity and security measures to stop propagation of the virus
(SD2) [49]	Public health officials should evaluate the measures to stop the virus	Social and sanitation measures should be rapidly modified depending on the seriousness of pandemic
(SD3) [47]	Decisions about testing should be implemented	Expanding testing capabilities is critical for slowing and controlling of the pandemic
(SD4) [51,53]	Hotels should inform the sanitation authorities in the case of infections	Hotels should develop protocols to alert the sanitation authorities
(SHM1) [54]	WHO protocols should be observed at the hotels	WHO protocols should be observed at the hotels and equally evaluated by official agencies for every hotel
(SHM2) [57,71]	Hotels should provide masks in preventing virus infection	The use of masks should be part of the health and sanitation protocol in hotels during the transition period
(SHM3) [58]	Workers should be the safest and the most hygienic	Sanitation protocols affecting work shifts, common spaces, rooms, and interaction with clients should be revised
(MT1) [60]	Government should have a strategy for testing and contact tracing of individuals	Updated tech should be available to hotels to assess worker and tourist protection
(MT2) [72]	Data collection is crucial to increase the available hospital capacity	Hotels should have a protocol about clients infected to be immediately communicated to hospitals
(MT3) [47]	Tech tools maximize public safety in similar scenarios	Companies should develop a strategy of using a comprehensive surveillance system between workers

**Table 6:** Loads.

	C19TP	MT	SD	SHM
C19TP1	0.889			
C19TP2	0.829			
C19TP3	0.861			
MT1		0.881		
MT2		0.796		
MT3		0.894		
SD1			0.851	
SD2			0.763	
SD3			0.892	
SD4			0.848	
SHM1				0.798
SHM2				0.887
SHM3				0.898

**Table 7:** Reliability, validity of the constructs.

Statistics	Cronbach Alfa	rho_A	CR	AVE	Fornell–Larcker Criterion			
Variables					C19TP	MT	SD	SHM
C19TP	0.824	0.825	0.895	0.740	0.860			
MT	0.820	0.823	0.893	0.737	0.513	0.858		
SD	0.860	0.872	0.905	0.705	0.612	0.426	0.840	
SHM	0.826	0.836	0.897	0.743	0.635	0.534	0.544	0.862

**Table 8:** Heterotrait–monotrait ratio (HTMT).

Variables	C19TP	SD	SHM	MT
C19TP				
MT	0.615			
SD	0.720	0.497		
SHM	0.759	0.642	0.637	

**Table 9:** Path coefficients.

Statistics/Variables	β	Lower CI	Higher CI	t Statistic	*p*-Value
MT → C19TP	0.177	0.105	0.258	4.527	0.000 ***
SD → C19TP	0.344	0.258	0.423	8.157	0.000 ***
SHM → C19TP	0.353	0.290	0.412	11.291	0.000 ***
SHM → MT	0.534	0.473	0.595	17.211	0.000 ***
SHM → SD	0.544	0.479	0.606	16.815	0.000 ***

Note: statistical significance: *** *p* < 0.001; n.s: not significant.

**Table 10:** Coefficient of determination (R^2^) and Stone–Geisser test (Q^2^).

Variables	Q^2^	R^2^
C19TP	0.381	0.526
MT	0.205	0.286
SD	0.204	0.296
SHM	--	--

**Table 11:** Hotel manager’s perception depending on the types of hotels.

	Beach Hotels	Rural Hotels	City Hotels	1 Star Hotels	2-Star Hotels	3-Star Hotels	4-Star Hotels	5-Star Hotels
C19TP	4.1	4.5	3.9	3.8	4.3	4.5	4.5	4.7
SD	4.5	4.8	4.2	3.9	4.0	4.6	4.7	4.6
MT	4.3	4.3	4.4	3.3	3.6	4.2	4.4	4.5
SHM	4.5	4.9	4.6	3.9	4.1	4.6	4.8	4.8
Total	203	386	153	56	122	240	199	125
